# Supplementary material for: Measuring Sleep Quality in the Hospital Environment with Wearable and Non-Wearable Devices in Adults with Stroke Undergoing Inpatient Rehabilitation
Source: Int J Environ Res Public Health. 2023 Feb 23;20(5):3984. doi: 10.3390/ijerph20053984 (PMC10001748; doi:10.3390/ijerph20053984)
Supplement: Supplementary file 1 [file ijerph-20-03984-s001.zip › ijerph-2131019-supplementary.pdf]

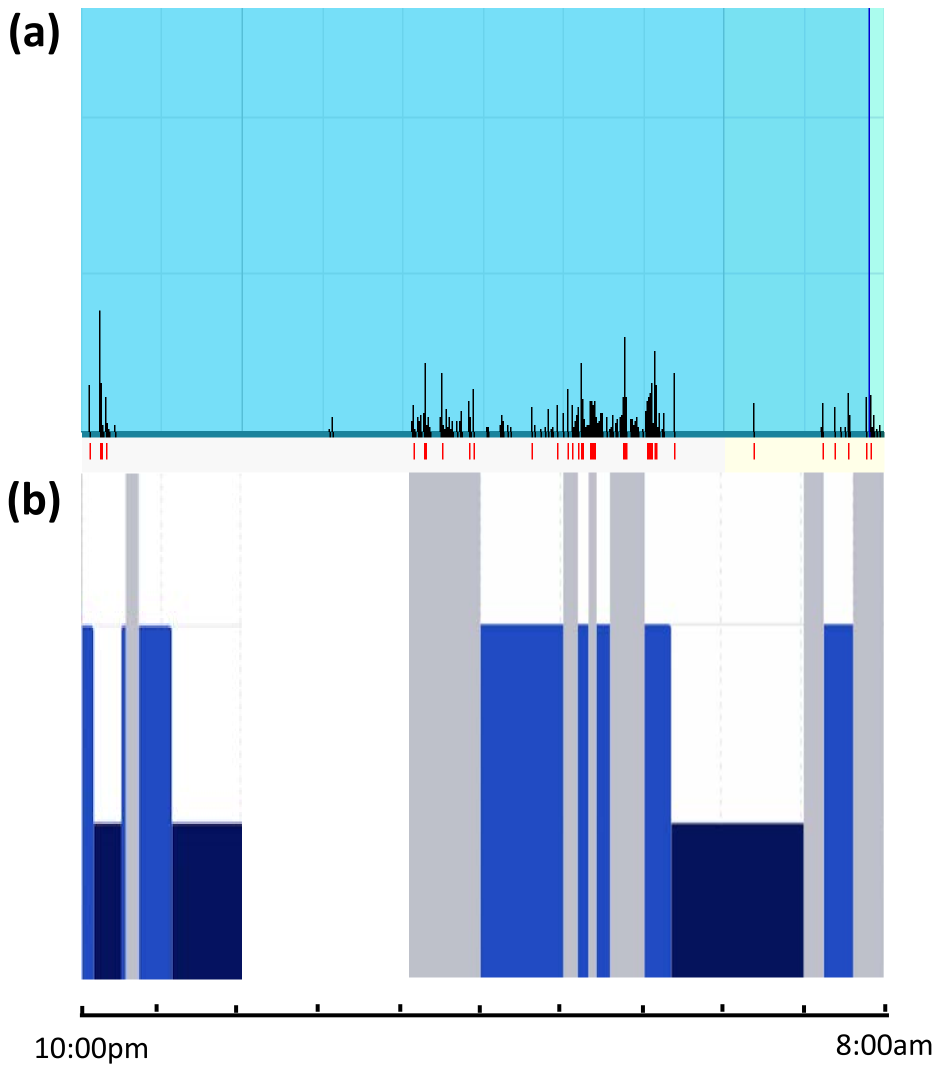

Figure S1. Graphical representation of sleep monitoring devices recording sleep activity across time for matched sub-sample participant one. (a) Philips actiwatch. Blue shaded region represents time asleep. Green shaded region represents resting in bed period. Black bar graph indicates movement and activity overnight. Red lines indicate awake periods. (b) WSA. Blue and black regions represent sleep and deep sleep periods respectively. Grey regions represent awake periods.

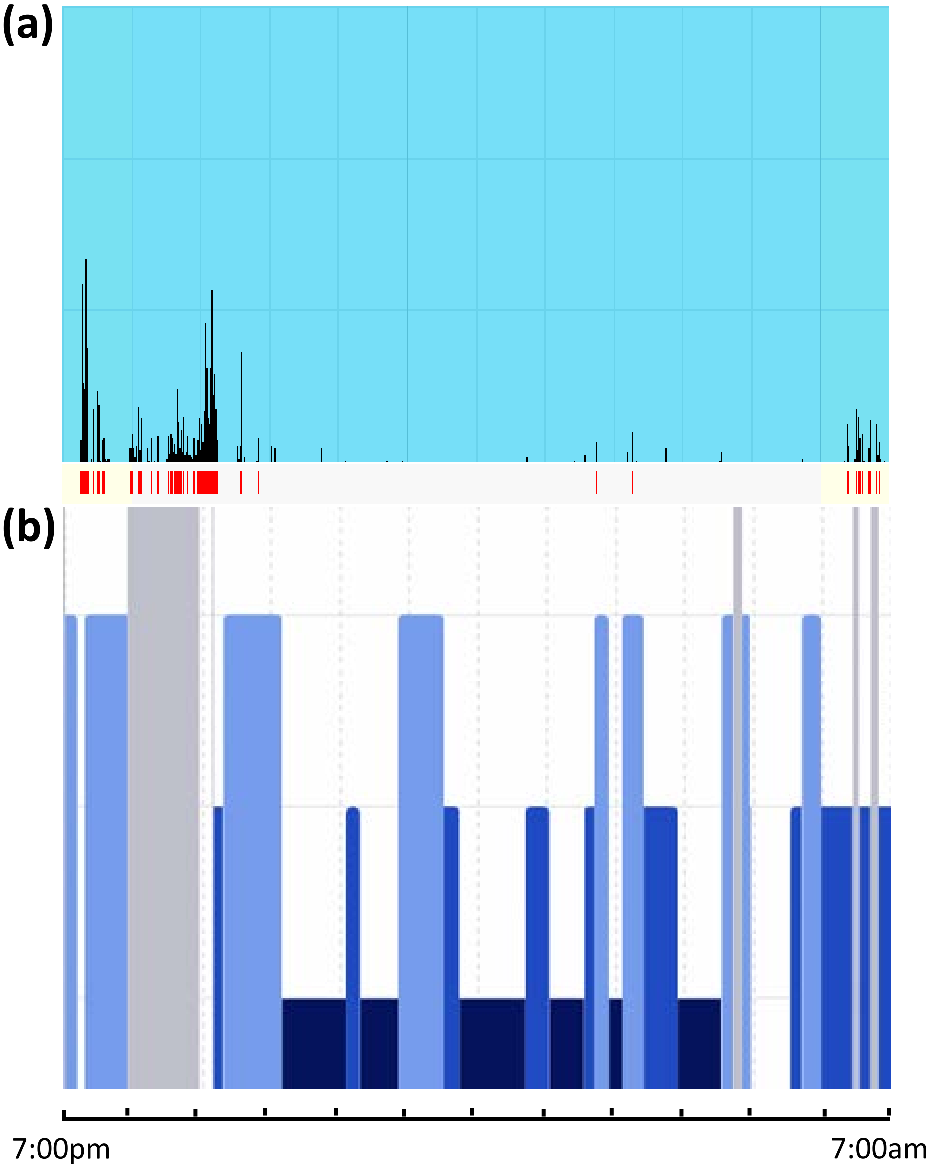

Figure S2. Graphical representation of sleep monitoring devices recording sleep activity across time for matched sub-sample participant two. (a) Philips actiwatch. (b) WSA.

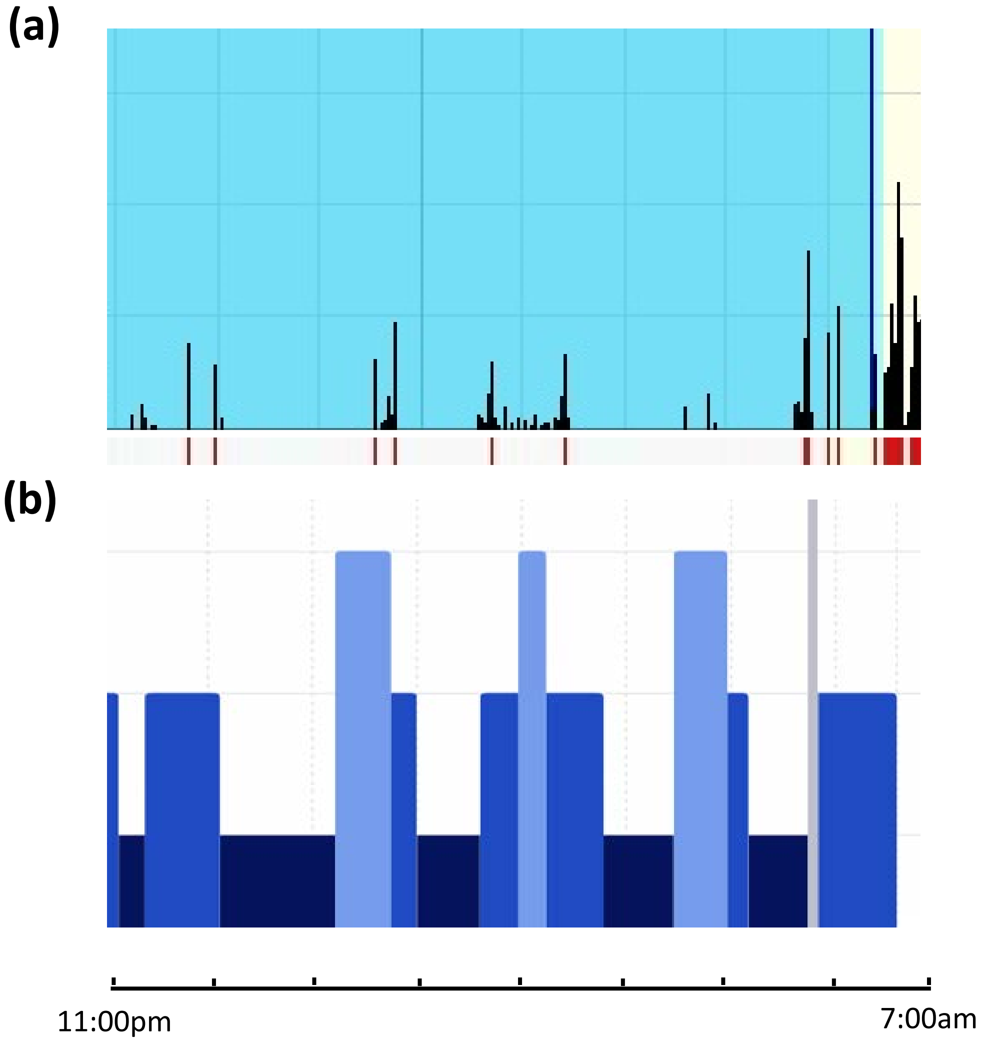

Figure S3. Graphical representation of sleep monitoring devices recording sleep activity across time for matched sub-sample participant three. (a) Philips actiwatch. (b) WSA.

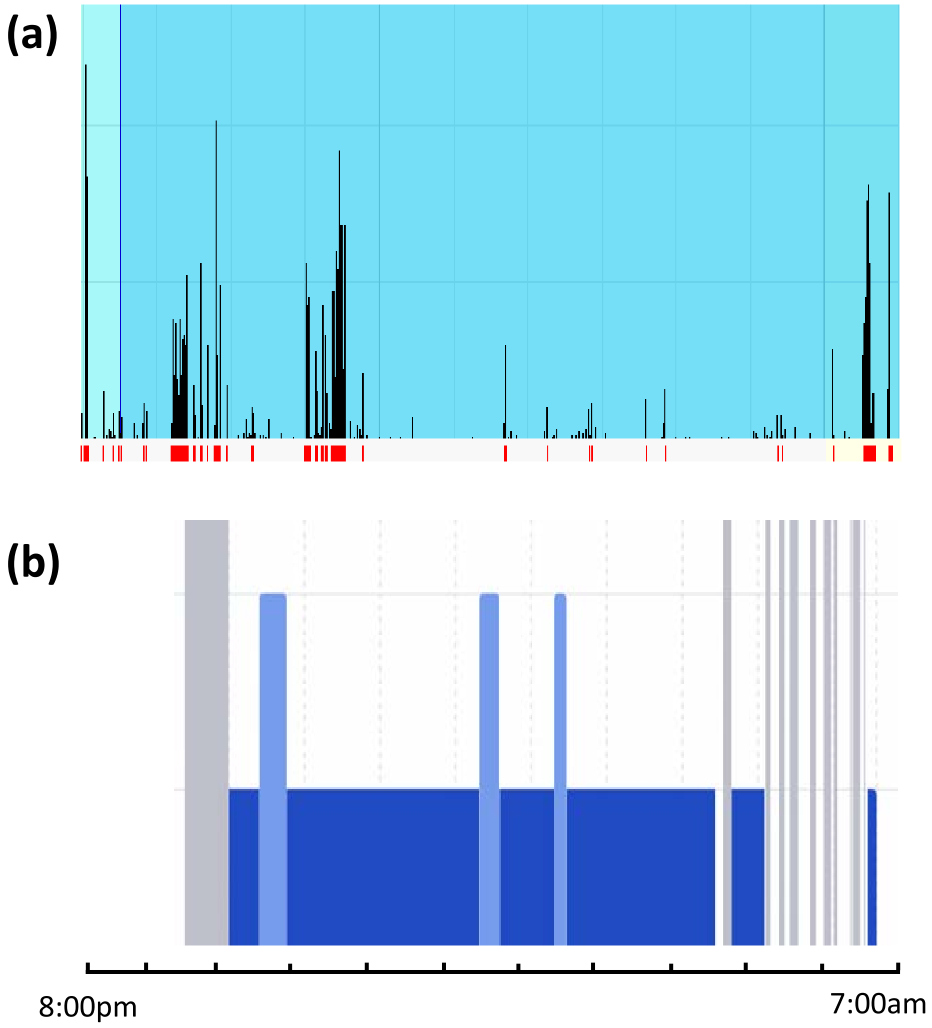

Figure S4. Graphical representation of sleep monitoring devices recording sleep activity across time for matched sub-sample participant four. (a) Philips actiwatch. (b) WSA.

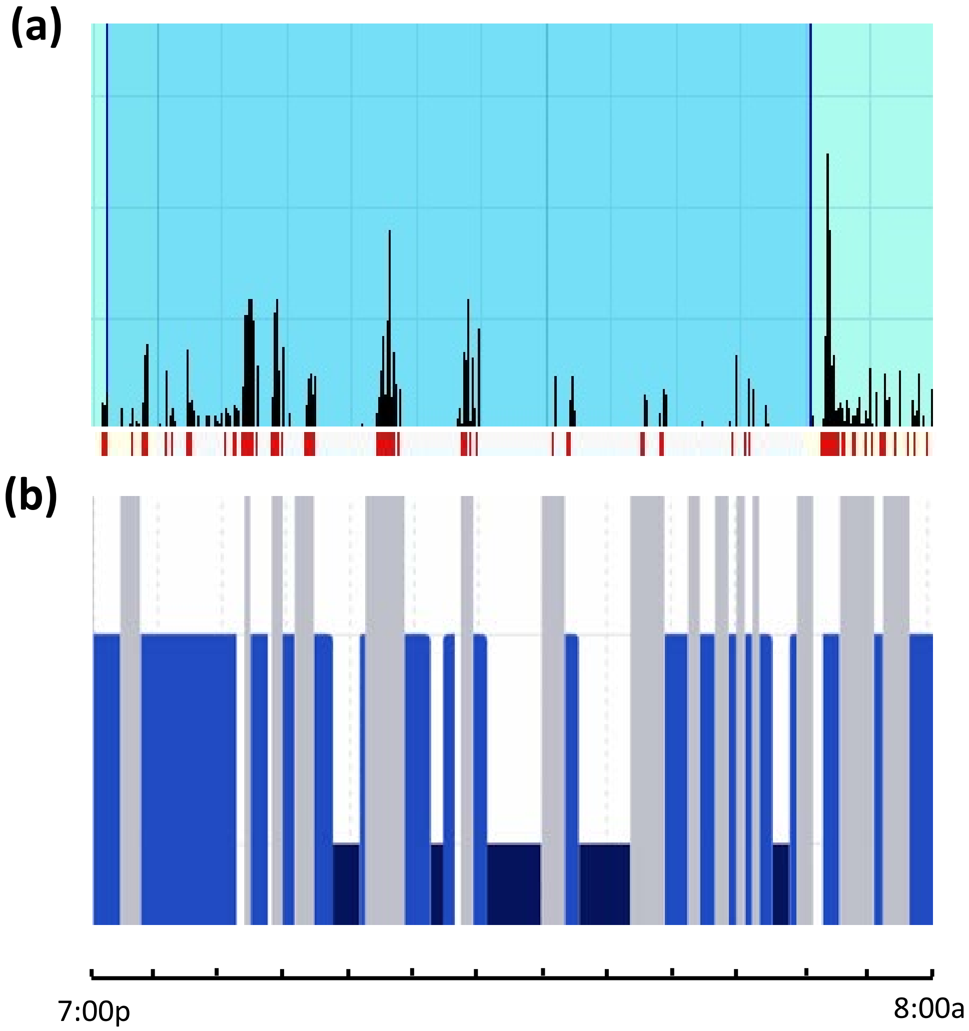

Figure S5. Graphical representation of sleep monitoring devices recording sleep activity across time for matched sub-sample participant five. (a) Philips actiwatch. (b) WSA.

Table S1. Measure and assessment of sleep quality for Philips actiwatch and WSA matched sub-sample participants (n=6), raw data.

Participant	Sleep Onset Latency *(mins)*			Total Sleep Time *(hrs)*			Awakenings *(count)*			Wake after sleep onset *(mins)*		Sleep Efficiency *(%)*	
	Actiwatch	WSA	Participant-reported	Actiwatch	WSA	Participant-reported	Actiwatch	WSA	Participant-reported	Actiwatch	WSA	Actiwatch	WSA
01	2.5	309	10	9.2	5.58	7.00	20	5	1	16	68	94.4	20.0
02	4.5	19	30	10.4	10.07	-	11	7	0	18	56	94.6	93.0
03	33	46	5	7.5	7.48	7.00	9	1	4	6.5	6	90.2	88.0
04	44	35	10	10.0	6.98	7.00	20	2	3	32	95	84.5	64.0
05	0	14	90	8.0	8.30	5.00	30	15	3	35.5	292	76.3	78.0
06	11.5	20	-	10.6	10.17	-	29	1	3	17.5	8	94.8	91.0

Abbreviations: hrs = hours; mins = minutes; SE = sleep efficiency; SOL = sleep onset latency; TST = total sleep time; WASO = wake after sleep onset; WSA = withings sleep analyzer
